# Where are Minors Initially Contacted for Sexual Exploitation Grooming in North America? Implications for Prevention Programming

**DOI:** 10.1177/15248380241290247

**Published:** 2024-10-15

**Authors:** Jeff Halvorsen

**Affiliations:** 1 University of Calgary, AB, Canada

**Keywords:** commercial sexual exploitation/human trafficking, sexual abuse, child maltreatment, prevention, child maltreatment, youth violence, Internet and abuse

## Abstract

Baird and Conolly’s systematic review on North American domestic minor sexual exploitation claimed that the literature most frequently identifies the internet as the initial site of contact. However, in my analysis of their sources, only three of the seven identified studies indicated that the internet was the site of initial recruitment, three studies did not have enough information to make a determination, and one study did not identify the internet as the site of initial recruitment. For the papers that did cite the internet as the initial site of recruitment, most identified the internet as among the least frequent locations of initial recruitment. The internet is not the most frequent location of initial recruitment and correcting this error is important because prevention programs that focus on raising awareness of risks may lead youth to develop a false sense of safety offline and contribute to the moral panic of youth risk online. Rather, prevention programs should focus on healthy relationship skills.

Sexual exploitation of minors is a serious global issue with long-term consequences (United Nations, Office of the High Commissioner of Human Rights, 2022). Patterns of trafficking differ across the globe with North American youth typically being groomed for domestic sexual exploitation as opposed to cross-border trafficking (Laird et al., 2023). Domestic minor sex trafficking (DMST) is the “recruitment, harboring, transportation, provision, or obtaining of a person for the purpose of a commercial sex act” within domestic borders, in which the person is a citizen or lawful permanent resident under the age of 18 (Trafficking Victims Protection Act [TVPA], 2000). Prevention of sexual exploitation of minors often involves education programs delivered to professionals who work with youth, caregivers, and youth themselves to increase awareness of warning signs and red flags (Jones et al., 2014). A common feature of these curricula is identifying the typical recruiters and locations of initial contact—for example, a male 2–3 years older, or someone posing as a friend online, and “people you meet online” (Finkelhor et al., 2021, p. 1236).

Understanding the site of initial recruitment is important for prevention and awareness programs. Finkelhor et al. (2021) argue that prevention programs that misrepresent where or how youth may be recruited can result in creating a false sense of security for minors with acquaintances and people with whom they have a romantic interest. While being prepared and cautious in one context, they may be unaware of the risk in another setting. Over the last 25 years, there has been a rising concern for adolescents’ safety online, particularly in terms of the risk of being groomed for sexual exploitation (Ali et al., 2023). As such, many prevention programs have been specifically designed to raise awareness of online risks for sexual exploitation. This raises the question of whether there is an emerging consensus in the peer-reviewed literature of where youth are most frequently contacted for grooming for sexual exploitation.

I was recently completing a literature review where I was examining this question, and after conducting my initial search, I found two excellent systematic reviews that took up this question. Baird and Connolly’s (2023) systematic review examined the recruitment and entrapment pathways of minors in the United States and Canada; the second review study was conducted by Finkelhor et al. (2021) and examined youth internet safety. More specifically, they examined how programming aligned with evidence.

Upon my initial reading, there appeared to be some tensions between the findings of Finkelhor et al. (2021) and Baird and Connolly’s (2023) systematic reviews. Finkelhor et al. (2021) suggested that there was no more risk online than in-person and that most youth are initially contacted in-person, followed by continued grooming online. On the other hand, Baird and Connolly (2023) suggested that of the 23 studies included, the “(m)ost commonly cited recruitment location [n=7; 30.4%] is online” (p. 193). This finding was supported by the following text:

initial contact via the internet was cited by more studies (*n* = 7) than other recruitment locations (Baird et al., 2020; Moore et al., 2020; J. E.O’Brien, 2018; J. E.O’Brien & Li, 2020; Rosenblatt, 2014; Tidball et al., 2016; Wells et al., 2012). Internet-facilitated recruitment occurs when traffickers access youth by frequenting online platforms popular with youth and using strategies such as initiating interpersonal relationships or even deceptively posing as an old friend ( J. E.O’Brien & Li, 2020; Rosenblatt, 2014). (Baird & Connolly, 2023, p. 194)

To investigate this apparent tension, I examined the source materials for both studies and came across what I believe to be an inaccuracy in Baird and Connolly’s (2023) findings. While they state their finding was supported by seven studies, upon my own reading of these source materials, I found that only three of these seven studies (Baird et al., 2020; Moore et al., 2020; Wells et al., 2012) explicitly referenced online as the site of initial recruitment.

The strongest evidence for the claim that initial contact online came from the lead author’s own study, Baird et al., (2020) in which they found that 64 (36%) of 233 cases began online. Of note, this study was an examination of police files, with data extraction of the files completed by police officers, and not data collected directly from victims of minor domestic sex trafficking. The second study was Moore and colleague’s (2020) examination of 25 medical records of minors, under 18, who had disclosed DMST. They reported that 3 (12%) were initially recruited over the internet. This was the least frequent location reported. The most frequent locations were the “home of the victim or a friend, 5(20%), [and] . . . at a social gathering 5(20%)” (Moore et al., 2020, p. 3153). The third study that identified online as the initial site of recruitment was a report by Wells et al., (2012) on the National Juvenile Prostitution Study that surveyed a sample of 2,398 law enforcement agencies in the United States. In this study, the authors collected law enforcement reports of 132 cases of juvenile sexual exploitation and while the authors did not systematically identify the location of initial recruitment, I identified two reports in the published article of where DMST recruitment began online, both in online chatrooms.

In my analysis, three (O’Brien, 2018; O’Brien & Li, 2020; Rosenblatt, 2014) of the seven studies required further information to interpret and confirm that the internet was the site of initial recruitment. In O’Brien’s (2018) study with 13 survivors of minor domestic sex trafficking, one paragraph referenced online recruitment:

a few individuals noted that interpersonal relationships formed via the Internet could increase risk of sexual exploitation. Examples of interpersonal relationships formed via the Internet included the Internet marketplace (e.g., Craigslist), Social media (e.g., Facebook), Internet-based dating sites (e.g., Tinder), and cyber bullying. (p. 7)

This referenced generalized increased risk and did not specifically note that any of the 13 participants were recruited for sexual exploitation through this location. Instead, they reported that “Almost all survivor participants were recruited via peer network (*n* = 11; 84.6%)” (p. 6). Yet, Baird and Conolly (2023) did not appear to highlight that this was a generalized risk and not a specific report.

O’Brien and Li’s (2020) study with 20 service providers reported that “almost all participants acknowledged that the internet played a huge role in facilitating DMST” (p. 194). This study did not include any reference to specific cases, but instead was focused on the perceptions of service providers. Another limitation is that this study was not a systematic examination of initial recruitment location, rather it was a study of the ways the internet is generally used in DMST.

Rosenblatt (2014), interviewed 37 survivors of DMST and reported on the vulnerabilities, deceptive recruitment strategies, and lures used to recruit victims to domestic minor sex trafficking. Access to the internet was noted as a vulnerability. Deceptive recruitment included two stories for the code of danger online. One participant shared:

I was online and they had my friend’s picture, a friend I haven’t talked to in a long time. So I talked to her and then a month later one of my other friends told me that she died in a car accident a long time ago. And this person knew everything about me. It was weird. I think it was him though. (p. 4)

This quote does not directly indicate that the site of online recruitment was a relationship that began online. The victim’s statement, “I think it was him though” (Rosenblatt, 2014, p. 4) indicates that there was not a direct connection between this online relationship and the site of initial recruitment from the perspective of the victim. The second quote supporting the code of “danger online” was an in vivo code of the words “danger online” taken from an interview. This was only referencing the concept of danger online, not reporting an experience. Baird and Conolly (2023) did not raise the limitations of these studies and the need for further information to confirm if individuals in these studies were initially recruited online.

I was unable to identify a reference to the internet as the site of initial recruitment for the remaining study (Tidball et al., 2016) that Baird and Conolly (2023) included. Tidball et al. (2016) reported that the internet was used for solicitation and buying sex from minors, but not as a location of initial recruitment. Overall, it appears that any reference to the internet or online in any form in these studies was included by Baird and Conolly (2023) in their count of the internet as the site of initial contact, thus overcounting these instances.

## Discussion

After my review, I concluded that only three of the seven studies cited supported the conclusion that the internet was the site of initial recruitment (Baird et al., 2020; Moore et al, 2020; Wells et al., 2012). I also found that three studies required further clarification (O’Brien, 2018; O’Brien & Li, 2020, Rosenblatt et al., 2014). As such, I would suggest that Baird and Conolly’s (2023) statement that “the internet is the most frequently cited location of initial recruitment” is misleading and in all but Baird et al. (2020), the internet was the least or near the least frequent location of initial recruitment (e.g., Moore et al., 2020; O’Brien, 2018; Wells et al., 2012).

Overestimating the online risk to youth has been critiqued in assessing relative risk youth experience. Finkelhor (2014) argued that online activity is no riskier than the in-person spaces youth inhabit and may even be safer. Consequently, O’Brien (2018) asserted that online relationships can be protective and foster resilience, particularly for vulnerable youth. In Finkelhor et al.’s (2021) systematic review of youth prevention programs suggested that many prevention programs focus on adult stranger online contact and create a false sense of safety for youth in their daily life. In the Finkelhor et al. (2021) analysis of the efficacy of prevention programs, they comment that “if they[youth] have communicated online with someone over time who seems interested in them, and with whom they have developed trust and perhaps shared images, a warning about ‘meeting up’ with someone who no longer seems a stranger will not be effective” (p. 1236). Accurate representation of where youth are at risk and how luring occurs is essential in providing effective prevention programming.

Overestimating online risk may also inadvertently contribute to an ongoing moral panic of youth and internet use (Walsh, 2020). Moral panic refers to “episodes where folk devils – moral outlaws . . . are blamed for societal malaise, the term captures how ‘right-thinking’ actors transmute deviant outsiders into potent sources of anxious indignation” (Walsh, 2020, pp. 840–841). The youth who are most at risk—those youth who experience accumulating intersectional oppressions—are often the ones who are later cast as risky by moral entrepreneurs seeking to maintain the innocence of childhood (Döring, 2009; Potter & Potter, 2001). Prevention programs that warn youth away from online activity because of the risk miss the opportunity to provide developmentally appropriate learning of healthy relationships and sexuality which will better prepare them for the risks faced both online and in-person (Finkelhor et al., 2020).
